# A New Stripline-Based Atmospheric Pressure Microwave Plasma Sheet Source Designed for Surface Modification of Materials

**DOI:** 10.3390/ma14237212

**Published:** 2021-11-26

**Authors:** Helena Nowakowska, Dariusz Czylkowski, Bartosz Hrycak, Mariusz Jasiński

**Affiliations:** Institute of Fluid Flow Machinery, Polish Academy of Sciences, Fiszera 14, 80-231 Gdańsk, Poland; helena@imp.gda.pl (H.N.); bhrycak@imp.gda.pl (B.H.); mj@imp.gda.pl (M.J.)

**Keywords:** microwave plasma source, plasma sheet, atmospheric pressure plasma, surface modification, tuning characteristics

## Abstract

A new type of microwave plasma source is presented in which plasma at atmospheric pressure is generated inside a quartz rectangular flat box placed in a stripline supplied by a 2.45 GHz coaxial line. The plasma has a sheet shape and is designed for surface modification. Electric field and power flux distributions, tuning characteristics, and power characteristics (ratios of radiated, absorbed, and entering power) are numerically studied for three configurations: open, semi-closed, and closed. The calculations show that near-zero radiation reduction is possible only for the closed configuration, while the ratio of radiated power to entering power is always greater than 30% for the other configurations. The moving plunger is not sufficient for the ratio of reflected to incident power to fall below 20% for both the closed and open configurations. This is possible for the semi-closed configuration, but then the radiated power is the highest. The experiment shows that for the same entering power, the plasma volume is largest for the closed configuration and smallest for the open configuration, which we attribute to the difference in radiated power. The plasma generated using the closed stripline configuration has a larger volume than plasma generated using the rectangular waveguide.

## 1. Introduction

Microwave discharges in gases under atmospheric pressure are usually generated in dielectric tubes [1] or in the ambient air at the extension of a metal line [2], and the produced plasma has the shape of a column, flame, or torch [3,4,5]. They are typically supplied from a coaxial line or—especially when higher powers are required—from a rectangular waveguide. An overview of the types of atmospheric-pressure microwave plasma sources (MPSs) and ways of supplying them can be found in [6]. Such discharges can be used for gas treatment or conversion, including: gas purification [7], decomposition of harmful gases [8,9], hydrogen production [10], CO_2_ decomposition [11], biomass gasification [12], and also for surface treatment [13], disinfection [14], etc.

A source of microwave plasma that is unusual in this respect is the microwave plasma sheet source (MPSS), which we developed and described earlier [15,16,17,18,19,20]. In this source, the plasma is produced in a box made of two parallel dielectric tiles closed on three sides. The box is placed in slots cut in the wider walls of a rectangular waveguide—perpendicular to the waveguide axis. The gas, which is introduced through two symmetrical inlets, is ionized in the microwave field and as a result plasma is generated. This plasma fills a large portion of the box and flows out of it through the open wall of the box. Plasma has the shape of the box and we dubbed it the plasma sheet. Due to its shape, such plasma is especially suitable for surface treatment, which have been successfully demonstrated for surface modification of polymeric materials such as polyethylene (PE) [21], polycarbonate (PC) [22], and poly(methyl methacrylate) (PMMA) [23] as well as for gradual etching of calcium carbonate crystals (CaCO_3_) [24].

A disadvantage of our plasma source is that it is quite large, bulky, and difficult to manipulate due to the waveguide used for the power supply which is rigidly connected to the microwave generator. To remove this drawback, we have modified the device by changing the power supply method from a rectangular waveguide to a stripline, which is supplied by a semi-rigid standard coaxial line. Striplines have already been used to power microwave plasma sources, but the plasma was produced in dielectric tubes and was column shaped [25,26]. The microstrip technology has also been used to generate plasma, but it was a microplasma of dimensions from μm to a few mm [27,28,29,30,31,32].

Replacing the waveguide with a stripline made our device lighter and more handy. It also allowed to follow the downscaling, the latest trend in engineering leading to the miniaturization of devices working simultaneously with lower energy consumption. The introduced changes significantly altered the design of the device, its electromagnetic (EM) characteristics, and the distribution of the EM field inside and outside it. A properly designed stripline, despite being an open structure, does not radiate EM waves into its surroundings, which has been demonstrated both theoretically and experimentally [25,26]. However, the presence of plasma (which is a strongly lossy conductor) in a stripline changes its properties. In such a structure, EM radiation is to be expected, as it is for other open microwave plasma sources [33]. Because our device is powered at several hundred watts, unwanted radiation may reach values that are harmful to human health [33,34]. To find ways to reduce this unfavorable effect and shield the radiation, we modified the standard stripline by placing additional metal plates on its open sides.

The purpose of this paper is to present the design of this novel stripline-based MPSS and investigate its basic properties. In particular, we study numerically the tuning characteristics of the device and EM radiation from it for three configurations (open—without shielding plates, semi-closed—with metal plates on one side, and closed—with metal plates on two sides). We examine also the power characteristics, i.e., the ratio of the power radiated to the power entering the MPSS device and the dependencies related to this ratio. We present and compare experimentally obtained plasmas for the three configurations for the same discharge conditions (the gas flow rate and the power entering the MPSS). We expect that this MPSS, like its predecessor, to be used for plasma surface modification of materials, and knowledge of the analyzed properties will enable its efficient application.

## 2. The Concept of the Stripline-Based MPSS

**Figure 1:**
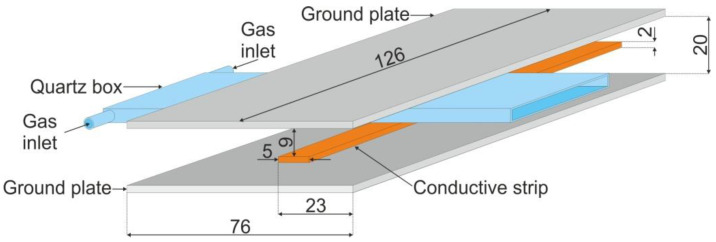
Schematic drawing of the stripline-based MPSS. Dimensions are in mm.

**Figure 2:**
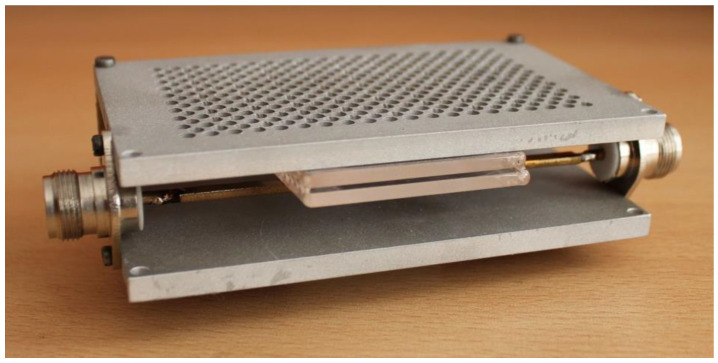
Photo of the stripline-based MPSS.

Schematic drawing of the stripline-based MPSS powered by microwaves at frequency *f* = 2.45 GHz is shown in Figure 1 while its photo is shown in Figure 2. For convenience, it is shown horizontally in the pictures, but it can work in different orientations. In the rest of this paper we show it with the box positioned vertically and the plasma flowing out the bottom. However, the reverse orientation, with the plasma flowing upwards, is also possible. The basic supply structure of the MPSS consists of two parallel ground plates with a conductive strip placed halfway between them. The conductive strip is not located symmetrically but it is displaced from the centerline of the ground plate. Both sides of the conductive strip are terminated with two N-type connectors to join it to the 50 Ω feeding coaxial line on one side and a short circuit on the other. The box in which plasma is generated is inserted perpendicular to the stripline. The box is made of two quartz rectangular tiles with a width, length, and thickness of 50 mm, 120 mm, and 3 mm, respectively. The tiles are arranged parallel to each other, and the distance between them is 1 mm. The space on the three sides of the tiles is closed. The other, narrower side of the box is open, allowing the gas and plasma to flow outward. The quartz box has a design the same as that presented in our previous work [19]. As it was shown there, to ensure uniform argon flow rate at the outlet of the box, its length should be greater than 100 mm and the gas flow rate should not exceed 20 L/min. The quartz box outlet extends 5 mm outside beyond the edge of the stripline’s ground plate. The working gas is delivered into the box via two opposite gas inlets parallel to the narrower walls of the box, located at the top of the box (shown in Figure 1). To initiate the plasma generation inside the box after supplying the plasma forming gas of the required flow rate and microwave power of sufficient enough level, a thin metal element can be introduced into the box (it is removed immediately after electrical breakdown in the gas). To visualize the plasma inside the MPSS, a number of holes with a diameter of 3 mm were made in the ground plate that is closer to the box, as can be seen in the photo presented in Figure 2. The diameter of the holes is small enough to be below cutoff of the 2.45 GHz frequency to prevent the electromagnetic radiation through these holes. All basic dimensions not provided in the above text are shown in Figure 1.

## 3. Numerical Analysis

### 3.1. Geometry

**Figure 3:**
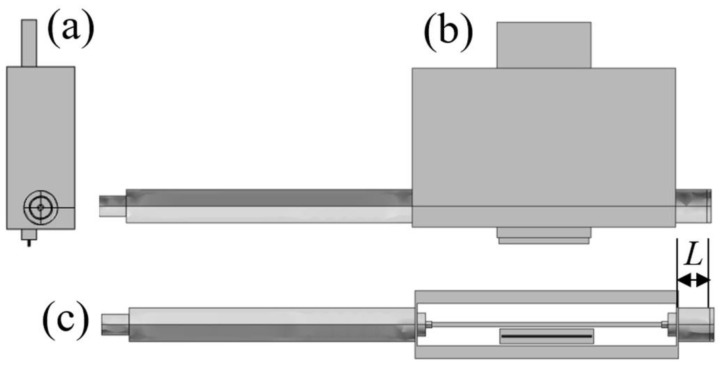
Three views of the MPSS model used for calculations, (**a**) left side view, (**b**) front view, and (**c**) bottom view. Position of the plunger is shown in (**c**).

The main purpose of the calculations is to determine the EM field distributions inside and outside the stripline-based MPSS and analyze the powers that characterize the EM field inducing the discharge. This enables understanding of physical phenomena and the optimal use of the device. The calculations are performed in 3-D geometry. The shape and dimensions reproduce the real device. Its three orthogonal views are shown in Figure 3. Only gas inlets made of dielectric are neglected because they introduce only minor disturbances to the analyzed EM field. A standard 50 Ω dielectric-filled coaxial line from which the device is fed is also included in the model. An identical coaxial line is on the opposite side of the device. This line is short-circuited at its end by a movable plunger. Its function is to improve the matching of the MPSS impedance to the feeding line. The distance of the plunger from the stripline wall is called the plunger position and designated as *L.*

**Figure 4:**
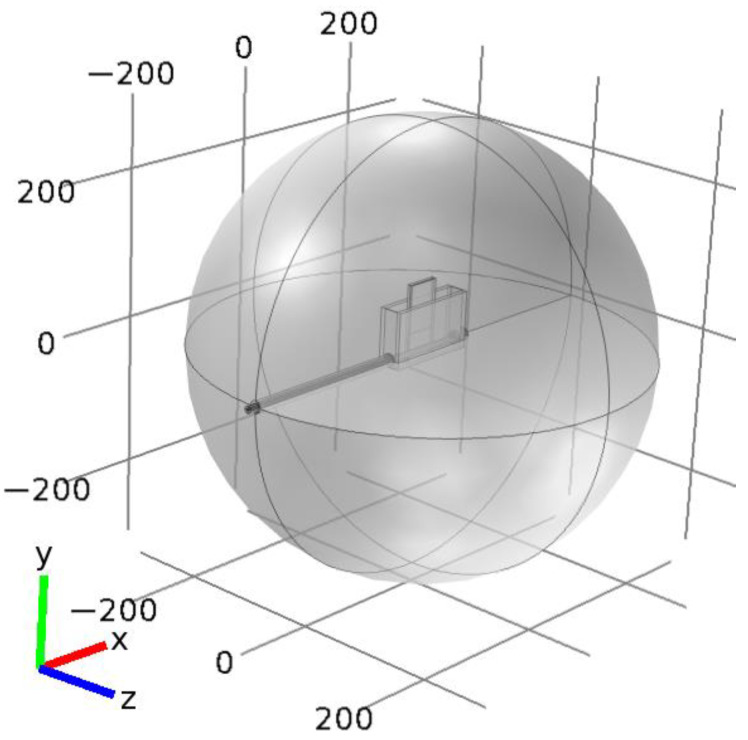
General view of the MPSS model in Cartesian coordinates with the radiation sphere.

The MPSS is an open structure, so EM radiation from it can be expected [34]. To take this fact into account in the calculations, it is assumed that the device is surrounded by an air-filled sphere representing the ambient space around the device (the radiation sphere), which is shown in Figure 4. The feeding coaxial line extends beyond the sphere, which allows for easy separation of the incident wave power from other components of the power balance.

### 3.2. Governing Equation and Boundary Conditions

The calculations are performed in a similar way as in our article [34] for TIAGO, another type of microwave plasma source. The electric component of the EM field in the calculation domain is determined from the vector wave equation, which follows directly from the Maxwell equations:

∇ × (∇ × **E**) − k_0_^2^ε_r_μ_r_**E** = 0, (1)

where **E** is a complex vector of electric field (phasor), ε_r_ is the relative complex permittivity of a medium, μ_r_ is the relative permeability of a medium (assumed 1 for all the media), k_0_ = ω/*c* is the wave number, ω = 2π*f* is the angular frequency, and *c* is the light velocity in vacuum. In the case when the medium of interest is plasma, the electric permittivity can be found from the Lorenz formula:

ε_p_ = 1 − *n*/(1 + *s*^2^) − *j ns*/(1 + *s*^2^), (2)

where *n* = *n*_e_/*n*_c_ and *s* = ν/ω are normalized electron density and electron-neutral collision frequency for momentum transfer, respectively, *n*_e_ is the electron density, *n*_c_ = *m*ε_0_ω^2^/*e*^2^ is the critical electron density, ν is the electron-neutral collision frequency for momentum transfer, *m* and *e* are the electron mass and charge, respectively, and ε_0_ is the free-space permittivity.

**Figure 5:**
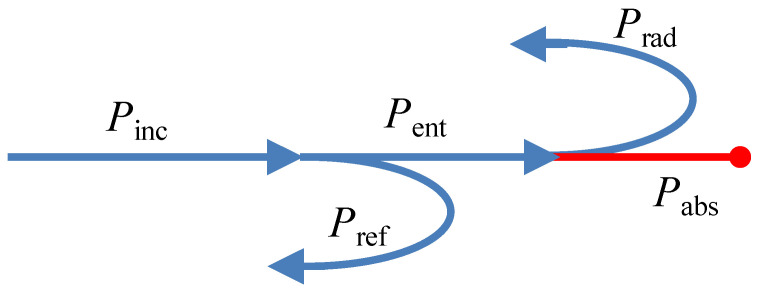
Power balance in the analyzed MPSS.

The power balance in the MPSS is shown schematically in Figure 5 and can be expressed as:

*P*_inc_ = *P*_ent_ + *P*_ref_ = (*P*_rad_ +*P*_abs_) + *P*_ref_, (3)

where *P*_inc_ is the incident wave power, i.e., the power carried by the EM wave from the microwave generator and delivered by the feeding line to the MPSS. Because the MPSS is a discontinuity in the feeding line, the wave is reflected, and only a part of the wave power enters the discharge. Power carried by the reflected wave and power entering the MPSS are designated as *P*_ref_ and *P*_ent_, respectively. The entering power is partly absorbed in the discharge (*P*_abs_) and partly radiated (*P*_rad_). The power *P*_inc_ is imposed in the calculation, the other powers are calculated.

The power absorbed in plasma (*P*_abs_) is determined as an integral of the electric power density *Q*_rh_ = (1/2) σ|**E**|^2^ over the plasma volume (*V*):

*P*_abs_ = ∫*_V_Q*_rh_ d*V*, (4)

where σ is the electrical conductivity of plasma and is related to the relative complex permittivity by the formula σ = ωε_0_ Im(ε_p_). The power radiated through the sphere (*P*_rad_) is the integral of the Poynting vector **S** = (1/2) Re(**E** × H*) over the sphere surface (*S*):

*P*_rad_ = ∫*_S_***S** d*S*, (5)

where **H** is the magnetic field component that can be found from **E** using Maxwell equations, and the asterisk indicates a conjugated number. Operators Re and Im determine the real and imaginary part of a complex number. The normal component of the Poynting vector is equal to the surface density of power flowing through a surface *W* (called also the power flux):

*W* = **S**·**n**, (6)

where **n** is the unit vector normal to the surface.

Perfect conductor conditions are imposed on all device metal walls. They imply that the electric field vector is normal to the surface. Scattering boundary conditions of spherical type are imposed on the radiation sphere. They imply that the surface of the sphere is transparent to the wave. The electric field vector is parallel and the Poynting vector is normal to the sphere surface for these conditions [35]. The feeding coaxial line is powered by the fundamental, transverse electromagnetic (TEM_00_) mode with an assumed power at the device input plane *P*_inc_. Equation (1) is linear with regard to **E**, therefore, with the assumed plasma parameters, the choice of *P*_inc_ values has no effect on the normalized electric field distributions and power ratios [34,36].

Calculations are performed for EM field frequency *f* = 2.45 GHz, the dielectric box relative permittivity ε_r_ = 3.8, the coaxial line filled with material of ε_r_ = 2.07 (polytetrafluoroethylene), the electron density in plasma *n*_e_ = 3.3 × 10^20^ m^−3^, and normalized electron-neutral collision frequency *s* = 5. Such plasma parameters can be expected from the measurements presented in the paper [19]. However, the results should be almost identical for other values, as long as the *n*_e_/*s* ratio is the same [34]. The height of the plasma sheet is—according to the experiment −40 mm and it protrudes 3 mm down from the dielectric box. The radiation sphere radius is 300 mm. The calculations were performed using commercial software COMSOL Multiphysics^®^.

## 4. Calculation Results

### 4.1. Open Configuration

**Figure 6:**
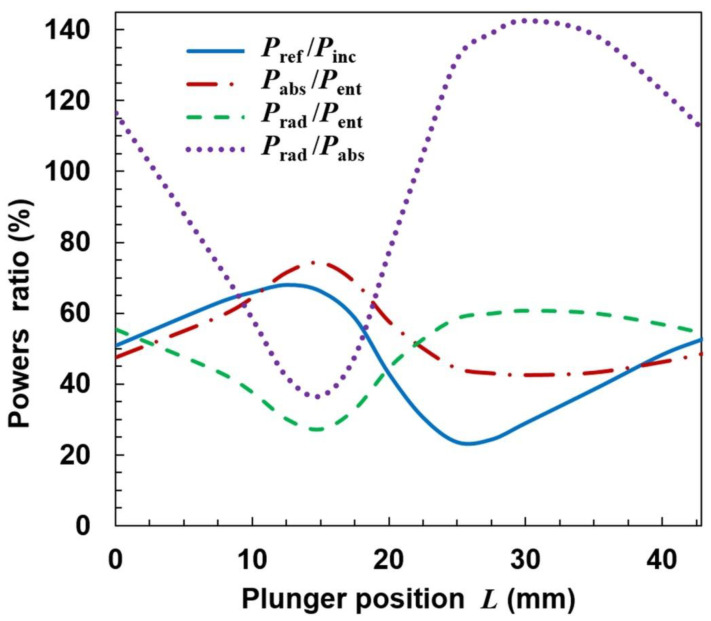
Dependence of powers ratios on the movable plunger position for open configuration and *n*_e_ = 3.3 × 10^20^ m^−3^ and *s* = 5.

**Figure 7:**
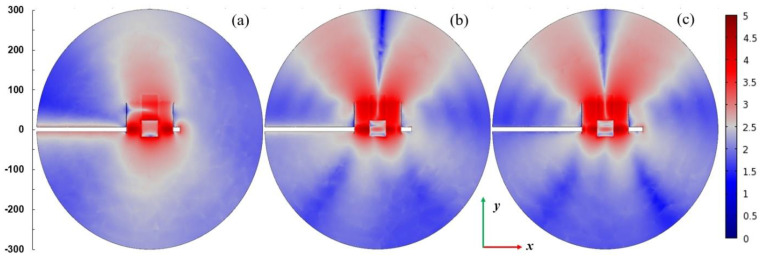
Spatial distributions of the log_10_ of |**E**|/(1 Vm^−1^) in *x*-*y* plane passing through the center of the plasma for *P*_ent_ = 100 W. (**a**) *L* = 15 mm, (**b**) *L* = 25 mm, and (**c**) *L* = 35 mm. The coordinate system is the same as in Figure 4. The scale on the left shows dimensions in mm.

**Figure 8:**
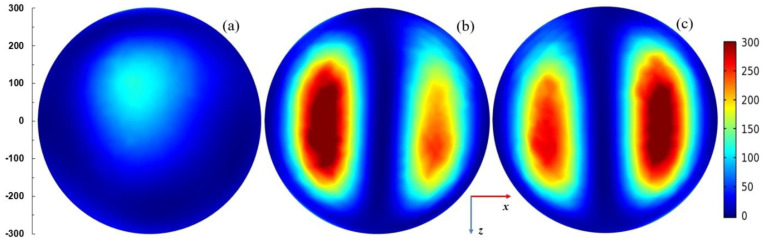
Distributions of the power flux *W*/(1 W/m^2^) on the upper part of the radiation sphere (with radius 300 mm) for *P*_ent_ = 100 W. (**a**) *L* = 15 mm, (**b**) *L* = 25 mm, and (**c**) *L* = 35 mm. The coordinate system the same as in Figure 4. The scale on the left shows dimensions in mm.

**Figure 9:**
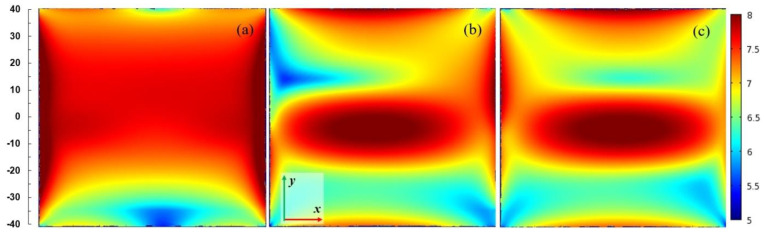
Spatial distributions of the log_10_ of *Q*_rh_/(1 Wm^−3^) in the *x*-*y* plane passing through the center of the plasma for *P*_ent_ = 100 W. (**a**) *L* = 15 mm, (**b**) *L* = 25 mm, and (**c**) *L* = 35 mm. The scale on the left shows dimensions in mm.

The results of calculations for the configuration shown in Figure 3 are presented in Figure 6, Figure 7, Figure 8 and Figure 9. Figure 6 shows how the power ratios change with the movable plunger position. The wavelength in dielectric-filled coaxial line is λ_d_ = *c*/(*f*ε_r_^1/2^), so in our case it is λ_d_ ≅ 85.1 mm. Due to formation of a standing wave (being the sum of the incident wave and the wave reflected from the plunger), the pattern in the figure repeats every λ_d_/2 = 42.9 mm. The relationship *P*_ref_/*P*_inc_ in the *L* function presented as the solid line is the so-called tuning characteristic [1,37], which is a measure of an MPS matching to the microwave track. (It is sometimes presented as the *P*_ref_/*P*_inc_(*L*/λ_d_) function). A value of *P*_ref_/*P*_inc_ = 100% means that the MPS does not match the track and the incident wave is reflected completely, which is a negative phenomenon. A value of *P*_ref_/*P*_inc_ = 0 means that the incident wave enters the MPS completely; it is a perfect match. A case when *P*_ref_/*P*_inc_ < 5% is considered to be a good match.

It is seen from Figure 6 that the reflected wave power depends on the plunger position and the ratio *P*_ref_/*P*_inc_ varies in the range from ~20% to ~70%. This means that perfect matching of the MPSS to the feeding line using one plunger is not possible in the presented configuration and other tuning means should be used to achieve good matching when carrying out an experiment. The ratio of the power absorbed to entering power (*P*_abs_/*P*_ent_) changes from ~40% to ~75%. The course of the *P*_rad_/*P*_ent_ variation is opposite to that of *P*_abs_/*P*_ent_, with values varying from ~25% to ~60%. The ratio *P*_rad_/*P*_abs_ may be up to 140%, with minimum value about 40%. These results are meaningful for interpretation of the experimental results also in other open MPSs. This is because the incident and reflected powers are usually measured, and it is their difference that is assumed to be the power absorbed in the plasma. Our calculations show that in an open system this is not true, because in a large range of *L* changes, the radiated power can be greater than the absorbed power, and the former (responsible for the plasma generation) can only be a fraction of the entering power. What is more, from Figure 6 it is seen that when the ratio *P*_ref_/*P*_inc_ is minimal (considered as good matching), the ratio *P*_rad_/*P*_abs_ is close to its maximal value. This means that the power that enters the MPSS is largely lost as the radiation to the surroundings and is not used for plasma generation. As could have been expected, the plasma extending beyond the ground plate causes strong radiation from the stripline, i.e., it significantly changes its properties [34].

Figure 7 presents spatial distributions of the electric field strength in logarithmic scale in the plane parallel to the plasma sheet and passing through the plasma center. The coordinate system is the same as in Figure 4. The power entering the MPSS is assumed 100 W. For *L* = 15 mm (when the radiated power is smallest), the electric field is confined in the vicinity of the MPSS. For *L* = 25 mm (when the radiated power is highest) and *L* = 35 mm, the electric field patterns above the MPSS are divided into two lobes. This effect is well seen in Figure 8 where the power flux on the upper part of the radiation sphere is presented. It can be noticed that the power flux is small for *L* = 15 mm (below 75 W/m^2^), whereas for *L* = 25 mm it achieves 300 W/m^2^ with two maxima, the larger of which is on the left side. For *L* = 35 mm the maximum is smaller and shifted to the right. It results from the figures that the MPSS radiates mainly towards the top.

Figure 9 shows the spatial distributions of the absorbed power density in logarithmic scale inside the plasma in the plane parallel to the plasma sheet and passing through the plasma center. For *L* = 15 mm (the case when the radiated to absorbed power ratio is smallest), it is more uniform than for the two other cases and the maxima are on the plasma edges. For *L* = 25 mm and *L* = 35 mm cases, the maximum of absorbed power density is along the strip of the MPSS. Maxima are also at the upper and lower borders of the plasma.

### 4.2. Semi-Closed Configuration

**Figure 10:**
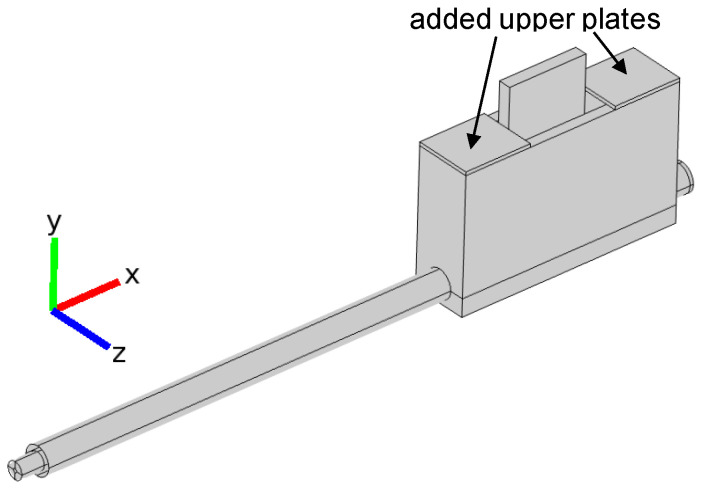
3D model of the MPSS with two upper metal plates added (semi-closed configuration).

Since we obtained from calculations that for an open configuration it is not possible to match the MPSS to the microwave track and that the radiated power cannot be reduced below 40% of the absorbed power, we modified our device by adding two 40 mm long metal plates to the top of the MPSS as shown in Figure 10, to check if this can reduce the radiation.

**Figure 11:**
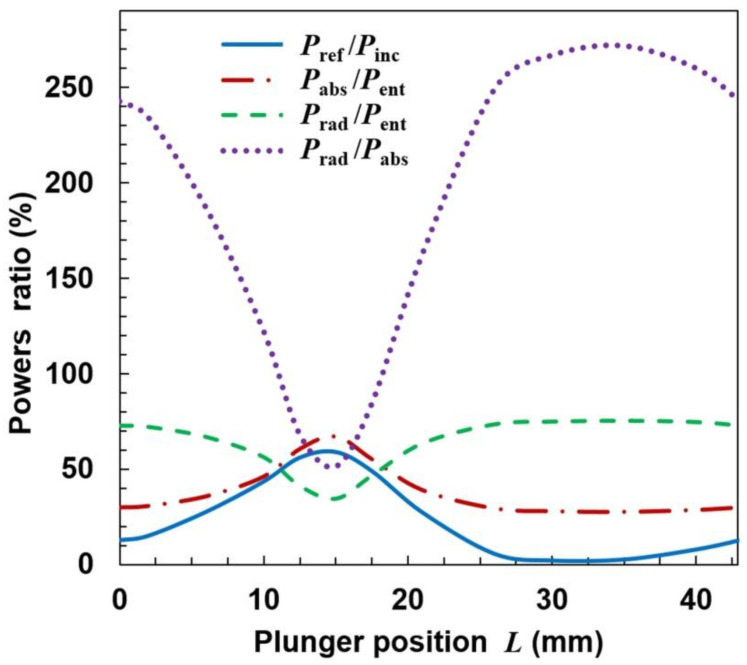
Dependence of power ratios on the movable plunger position for semi-closed configuration and the same plasma as in Figure 6.

**Figure 12:**
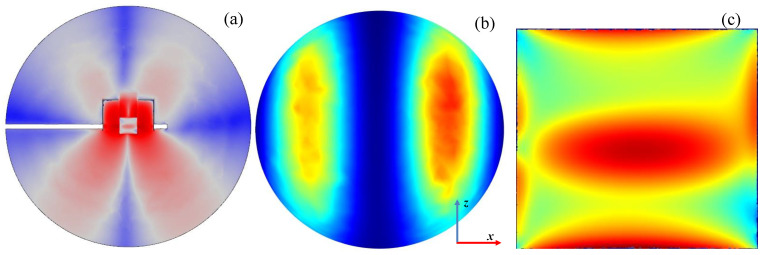
(**a**) Distributions of the log_10_ of |**E**|/(1 V/m); (**b**) distributions of the power flux *W*/(1 W/m^2^) on the lower part of the radiation sphere; and (**c**) distributions of the log_10_ of *Q*_rh_/(1 Wm^−3^). Details in (**a**–**c**) are the same as in Figure 7, Figure 8 and Figure 9, respectively. *P*_ent_ = 100 W and *L* = 35 mm.

The calculations show that such modification does not prevent radiation. On the contrary, for some plunger positions the radiation can be increased. It is seen in Figure 11 that the ratio *P*_ref_/*P*_inc_ varies in the range of ~0–~60% with the change of *L*, i.e., that the reflected power can be reduced almost to zero. However, when *P*_ref_/*P*_inc_ is close to zero, the maximum radiation is observed (*L* ≅ 35 mm), and the *P*_rad_/*P*_abs_ goes up over 250% (with minimum value ~50%). This means that the reduction of the reflected wave occurs due to the increase in radiation, and not the power absorption in the plasma, which is a negative phenomenon. Spatial distributions of quantities related to the electromagnetic field for the case with maximal radiation are shown in Figure 12. The electric field distributions in Figure 12a show that the addition of plates causes the wave to reflect from them and changes the direction in which the device radiates. There are two strong downward-facing lobes and two weak upward-facing lobes in the electric field. The strong lobes can also be seen in the power characteristic shown in Figure 12b, where the power density on the lower part of radiation on the sphere is shown. Similarly as for the open configuration, the absorbed power density is maximal along the strip, which is seen in Figure 12c.

### 4.3. Calculation Results for Closed Configuration

**Figure 13:**
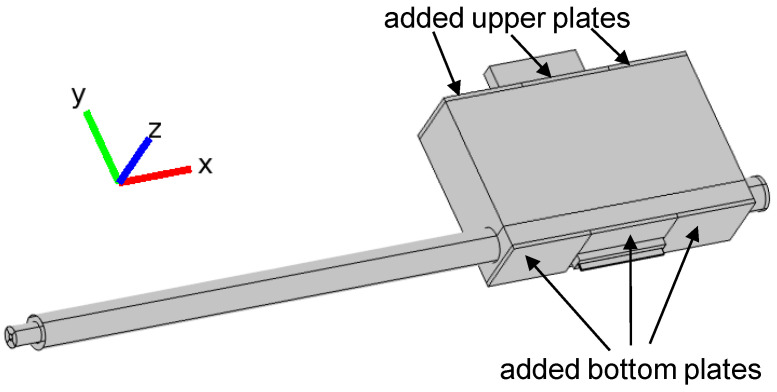
3D model of the MPSS with added upper and bottom plates (closed configuration).

As the semi-closed configuration proved no more advantageous than the open configuration, we performed calculations for a configuration with additional plates also at the bottom, resulting in a closed configuration, seen in Figure 13. In this configuration, only the quartz box and plasma extend beyond the boundaries of the ground plates.

**Figure 14:**
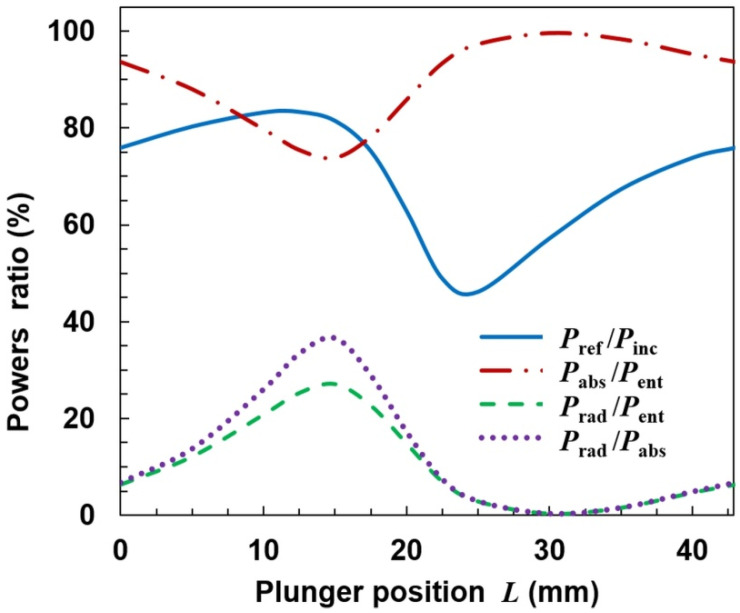
Dependence of power ratio on movable plunger position for closed configuration.

**Figure 15:**
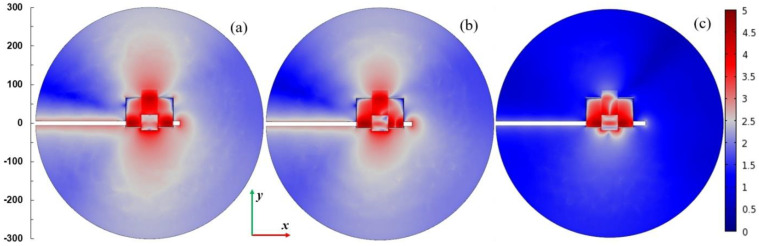
Spatial distributions of the log_10_ of |**E**|/(1 Vm^−1^) in *x*-*y* plane passing through the center of the plasma for *P*_ent_ = 100 W and closed configuration. (**a**) *L* = 15 mm, (**b**) *L* = 20 mm, and (**c**) *L* = 30 mm. The scale is the same as in Figure 7. The scale on the left shows dimensions in mm.

**Figure 16:**
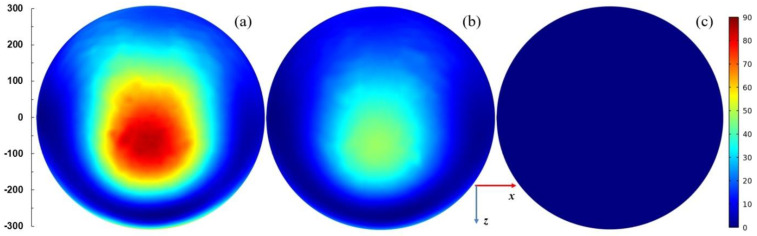
Distributions of the power flux, *W,* on the top part of the radiation sphere (with radius 300 mm) for *P*_ent_ = 100 W and closed configuration. (**a**) *L* = 15 mm, (**b**) *L* = 20 mm, and (**c**) *L* = 30 mm. The scale is the same as in Figure 8. The scale on the left shows dimensions in mm.

**Figure 17:**
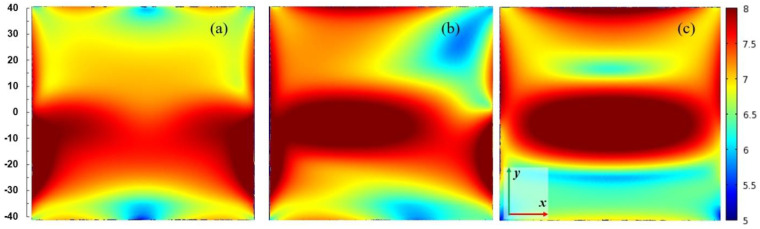
Spatial distributions of the log_10_ of *Q*_rh_/(1 Wm^−3^) in *x*-*y* plane passing through the center of the plasma for *P*_ent_ = 100 W and closed configuration. (**a**) *L* = 15 mm, (**b**) *L* = 20 mm, and (**c**) *L* = 30 mm. The scale is the same as in Figure 9. The scale on the left shows dimensions in mm.

Figure 14, Figure 15, Figure 16 and Figure 17 show results of calculations performed for the closed configuration. It is seen from Figure 14 that the radiation (the dashed line) cannot be minimized to zero over the entire range of the plunger position, which is due to the fact that the plasma extends beyond the boundaries of the plates. However, it can be reduced to less than 5% of the entering power in quite a wide range of the plunger position and is always lower that the absorbed one. The power reflected varies from ~46% to ~83% of the incident power. This means that additional tuning means are necessary to minimize it to acceptable values in the experiment. Figure 15 shows the electric field strength distribution for three cases including minimal (*L* ≅ 30 mm) and maximal radiation (*L* ≅ 15 mm) cases. It is worth noting that when the system radiates, its metal elements also (such as the plunger) become a source of radiation because electric currents are induced on them. In the closed configuration for the maximal radiation case, only one lower and one upper lobe are observed in the electric field patterns. This effect is also seen in Figure 16, where the energy flux over the top hemisphere is presented. The maximal power flux about 90 W/m^2^ is for *L* = 15 mm. The distribution of the absorbed power density is presented in Figure 17. The maximum is along the strip and for the case with minimal radiation (*L* = 30 mm) its distribution is almost symmetrical.

**Figure 18:**
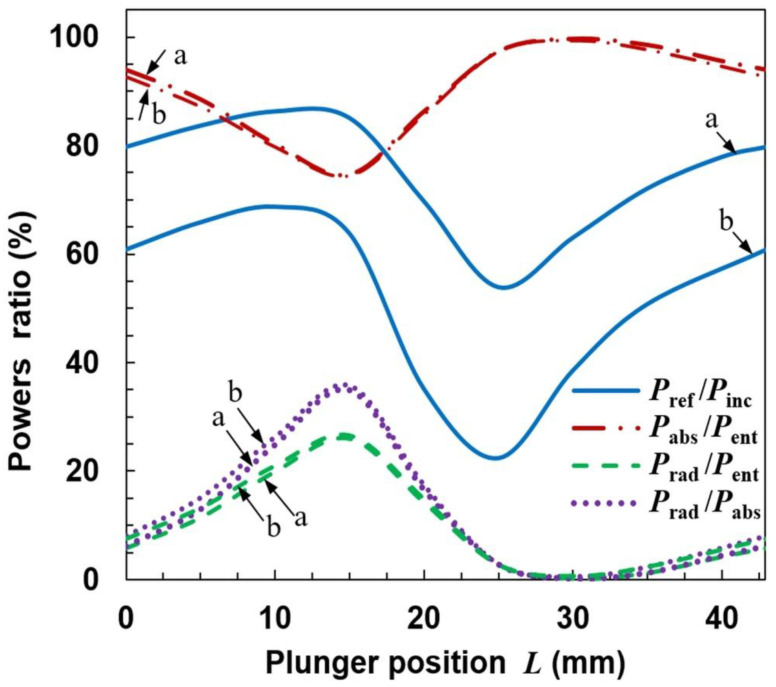
Dependence of powers ratio on movable plunger position for closed configuration, *s* = 10 and two values of the electron density. a: *n*_e_ = 1 × 10^20^ m^−3^ and b: *n*_e_ = 1 × 10^21^ m^−3^.

Although the calculations presented above refer to the assumed plasma properties, we have checked that the general properties of the solutions are similar for other parameters as well. For example, the power analysis for closed configuration, *s* = 10 and two values of *n*_e_ (1 × 10^20^ m^−3^ and 1 × 10^21^ m^−3^) is shown in Figure 18. Such parameters correspond to different absorbed powers than in the previous case. The courses of *P*_abs_/*P*_ent_, *P*_rad_/*P*_ent_ and *P*_rad_/*P*_abs_ for both values of *n*_e_ are almost the same as those presented in Figure 14. The shape of the tuning characteristics (*P*_ref_/*P*_inc_ as *L* function) is similar to that in Figure 14 but the values are lower for the lower *n*_e_ and higher for the higher *n*_e_. In no case can the reflected wave power be reduced to zero.

Calculations show that a typical stripline, when a quartz box with plasma is placed in it, begins to radiate strongly. To avoid this, the parallel ground plates must be supplemented with additional metal plates to form a metal box through which only the quartz box protrudes. With such a design, the radiation can be reduced to zero for some plunger positions. However, even with this design, radiation can be noticeable (up to 20% of the power entering) for other positions of the plunger because the plasma extends outside the box.

Calculations further show that it is not possible to match a closed MPSS to a microwave track using a single movable plunger. Since the value of the reflected power exceeding 5% of the incident power is unacceptable, the microwave system must be supplemented with additional tuning elements. It may be, for example, a three-stub tuner placed before the MPSS.

## 5. Experiment

### 5.1. Experimental Arrangement

**Figure 19:**
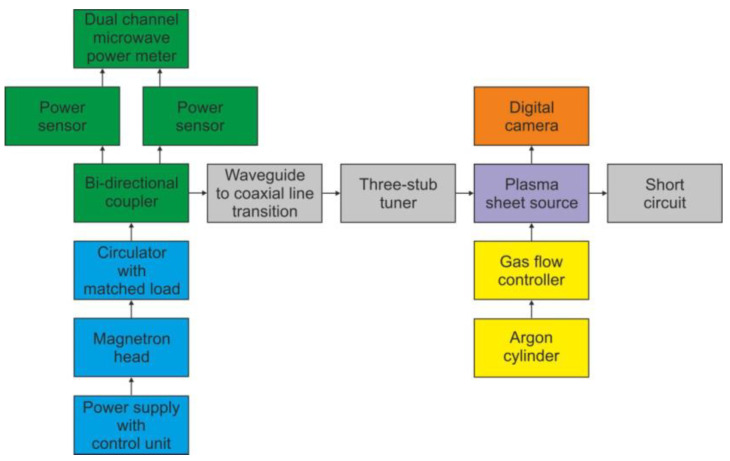
Schematic diagram of the experimental arrangement with the atmospheric pressure microwave argon plasma sheet source.

To examine the operation of the stripline-based MPSS, we conducted an experimental analysis of it in three configurations (open, semi-closed, and closed). Since calculations show that the moving plunger cannot be an effective tuning tool, to simplify the construction, a fixed plunger was used as a short circuit on the side opposite the supply line (*L* value was 30 mm). All configurations were tested on the same experimental setup, the schematic diagram of which is shown in Figure 19. Microwaves were produced by a SAIREM GMP 20 KE/D microwave generator that consists of the high-voltage power supply with control unit, magnetron head and circulator with matched load. The generator was operated at a frequency of 2.45 GHz in a continuous mode with controllable output microwave power. A WR-340 rectangular waveguide, operating in the fundamental TE_10_ mode was used to transmit the power from the generator. For sampling the microwave signal for both the incident and reflected wave, a bi-directional coupler was used. The fractions of the microwave power, diverted by the coupler, were measured with two power sensors connected with a dual-channel power meter. The microwave power entering the MPSS was calculated as *P*_ent_ = *P*_inc_ *− P*_ref_ (see Figure 5). A waveguide-to-coaxial line transition of standing wave ratio (SWR) < 1.09 (<−27 dB) was used to enable feeding the device from a 50 Ω dielectric-filled coaxial line. To improve impedance matching and minimize the reflected power, the MPSS was preceded by a coaxial three-stub tuner. The plasma forming gas (Ar, 99.998% vol., Air Liquide Poland) was delivered to the quartz box from the compressed gas cylinder and the gas flow rate was set by the Bronkhorst thermal mass flow controller of EL-FLOW series. A CANON EOS 550D digital camera was used to perform the plasma visualization.

### 5.2. Experimental Results

All experimental tests of the presented MPSS were performed at an argon flow rate of 15 L/min and the microwave power entering the device was 250 W. As mentioned earlier, the first parameter results from previous observations related to the uniformity of argon flow at the outlet of the quartz box [19], while the second is related to the limitations of the coaxial elements used in the experimental setup (using powers higher than 300 W can damage them).

**Figure 20:**
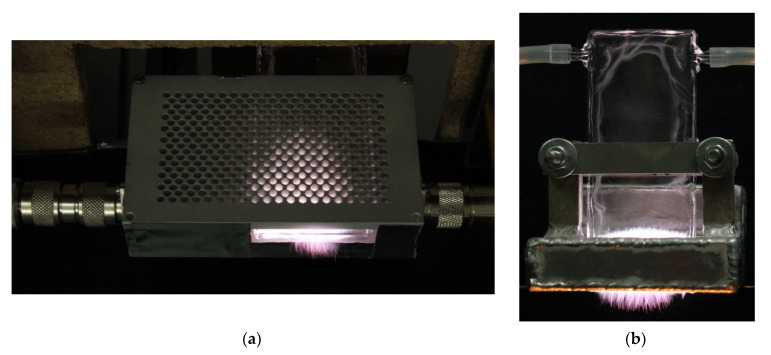
Plasma visualization for: (**a**) stripline-supplied closed configuration and (**b**) waveguide-supplied configuration. Microwave power entering the device 250 W, argon flow rate 15 L/min.

Figure 20a shows the MPSS in a stripline-based version (analyzed in this paper) and, for comparison, Figure 20b shows a waveguide-based version (analyzed in [19]) at the same conditions. As one can see, the generated plasmas differ in shape and dimensions. In the former case, the height of the plasma from the bottom edge of the box is about 48 mm and is greater than in the latter case, where it is about 30 mm. This is due to the difference in the distribution of the electric field in these two plasma sources. In the case of the waveguide, the field is limited by its wider walls (whose distance is 17 mm), while in the case of the stripline, the field is limited by ground plates (whose width is 76 mm). The plasma below the box outlet is of similar height in both cases, although for the waveguide-supplied one it is more uniform. In each case, fluctuating filaments are visible both in the plasma inside the box and at the outlet. Such filamentary plasma structure is typical for the radio and microwave frequency argon discharges at atmospheric pressure and is related to the electromagnetic skin effect, non-uniform gas heating, presence of molecular ions, and electron energy distribution function [38,39,40,41,42].

**Figure 21:**
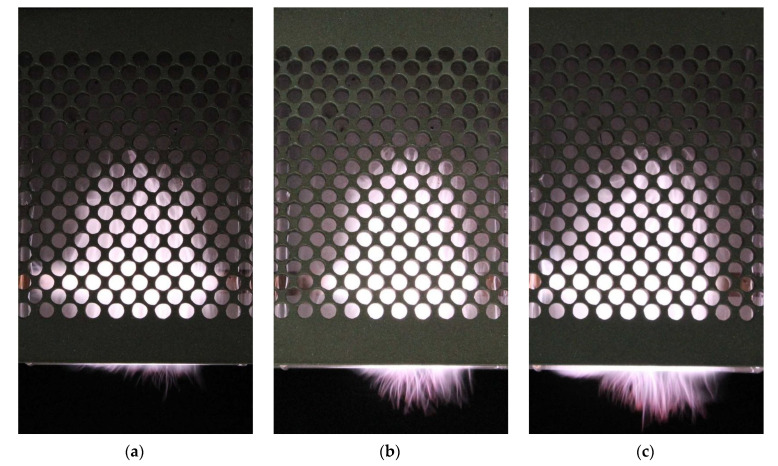
Plasma visualization for: (**a**) open (*P*_ref_/*P*_inc_ = 25%), (**b**) semi-closed (*P*_ref_/*P*_inc_ = 15%), and (**c**) closed (*P*_ref_/*P*_inc_ = 40%) configuration. Microwave power entering the MPSS is 250 W, argon flow rate 15 L/min.

Figure 21 shows the photos of the plasma for open, semi-closed, and closed configurations of the stripline-supplied MPSS. Matching of the device to the microwave track was achieved using the three-stub tuner. In the open configuration, the plasma has the smallest volume. Its height above the gas outlet is the smallest and in addition the height below the outlet is significantly smaller. In the closed configuration, the plasma has the largest volume and shines the brightest. The filaments protrude the greatest distance. Its width at the gas outlet is also the greatest. Since the entering power and the gas flow rate are the same, we attribute differences in plasma volume to differences in radiated power. It is highest in the open configuration and lowest in the closed configuration. The power absorbed and therefore suitable for plasma generation is the largest in the closed configuration.

## 6. Summary and Conclusions

The paper presents a new type of microwave plasma source that is stripline-based and supplied by a standard coaxial line. It produces atmospheric pressure sheet-shaped plasma in a dielectric box and is intended for surface modification of materials. This device is lighter and more handy than the one using a rectangular waveguide.

We numerically investigated the electric field distributions inside and outside the device and the power relations for assumed plasma parameters and its three configurations: open, semi-closed (with shielding metal plates at upper part), and closed (with shielding metal plates at upper at bottom part). Calculations show that for an open configuration, it is not possible to bring the reflected and radiated power levels below acceptable values using only a moving plunger. The levels are always above 20% of the incident and entering powers, respectively. Additionally, when the reflected wave power is the smallest, the ratio of radiated power to entering power exceeds 70%, which means that the input power is mainly lost as radiation and is not used for plasma generation.

The same unfavorable effect is for the semi-closed configuration. Although it is possible to reduce the reflected power to almost zero by means of the moving plunger, the radiated power is then the highest. It is not possible to reduce the radiated power to entering power ratio below 40%.

Reducing the radiation to almost zero is possible only for the closed configuration, though not in the entire range of the plunger position. This is due to the fact that the plasma also partially protrudes from the device in this situation. However, the range of plunger position, in which the ratio of radiated power to entering power is less than 5%, is wide enough for the device to operate efficiently.

The plasmas generated in the experiment in the three configurations differ in volume and shape for the same discharge conditions (entering power 250 W and gas flow rate 15 L/min). For the closed configuration, the plasma volume is the largest and it shines the brightest. For the open configuration, the plasma volume is the smallest. This confirms our calculations that the classic open stripline is not a viable solution and that in order to improve the energy efficiency of the device it is necessary to enclose it with metal plates that shield the radiation.

The experiment also shows that the plasma sheet generated using a stripline in the closed configurations has a larger volume than that produced using a rectangular waveguide, but extends less outside the box. We attribute this to differences in the distribution of the electric field in the stripline and the waveguide.

As it has already been emphasized in this paper, the presented new stripline-based MPSS is dedicated to plasma modification of various material surfaces. The numerical and experimental studies are a step towards understanding its basic properties necessary for its efficient application. The presented MPSS is expected to be as promising as its waveguide-based predecessor. In order to determine this, experimental studies concerning plasma surface modification of selected polymers are our future goals.

## Data Availability

The data presented in this study are available on request from the corresponding author.
